# Food supplementing peregrine falcon (*Falco peregrinus tundrius*) nests increases reproductive success with no change in mean parental provisioning rate

**DOI:** 10.1098/rsos.240576

**Published:** 2024-09-25

**Authors:** Rebekah A. McKinnon, Erik Hedlin, Kevin Hawkshaw, Kimberley J. Mathot

**Affiliations:** ^1^ Department of Biological Sciences, University of Alberta, CW 405, Biological Sciences Building, Edmonton, Alberta T6G 2E9, Canada; ^2^ Nunavut Wildlife Cooperative Research Unit, University of Alberta, CW 405, Biological Sciences Building, Edmonton, Alberta T6G 2E9, Canada; ^3^ Department of Renewable Resources, University of Alberta, GSB 751, Edmonton, AB T6G 0N4, Canada; ^4^ Department of Biological Sciences, Canada Research Chair in Integrative Ecology, University of Alberta, Edmonton, Alberta T6G 2E9, Canada

**Keywords:** parental care, food supplementation, experiment, life-history trade-offs, reproductive success

## Abstract

Parents are expected to exhibit intermediate levels of investment in parental care that reflect the trade-off between current versus future reproduction. Providing parents with supplemental food may allow for increased care to the current brood (additive model), re-allocation of parental effort to other behaviours such as self-maintenance (substitution model), or may provide parents with a buffer against provisioning shortfalls (insurance model). We investigated the impact of parental food supplementation on provisioning behaviour and breeding success in Arctic-breeding peregrine falcons (*Falco peregrinus tundrius*) over five successive breeding seasons (2013−2017). We found that supplemental feeding had no impact on mean provisioning rates, yet resulted in increased nestling survival probability, increased nestling body mass and decreased variance in nestling body mass and provisioning rates. These results are consistent with parents adopting a hybrid of the additive and substitution models. We suggest that food supplementation enables increased investment in other forms of parental care (e.g. nest defence, brooding) without altering mean provisioning rates. The lack of observed effects on mean provisioning rates, coupled with increased survival and body mass of offspring, suggests a potential reallocation of parental effort. The findings contribute to understanding the responses of peregrine falcons to food supplementation, highlighting the need for future studies to explore broader environmental contexts and potential long-term effects on parental survival and future reproduction.

## 1. Introduction

Food is often a key factor limiting reproductive success in birds (reviewed in Martin [1]). Greater food availability generally increases current reproductive success [2,3]. While it seems logical that greater food availability should allow parents to provide a greater level of care to current offspring, thereby increasing reproductive success [3–5], studies that have manipulated food availability (i.e. food supplementation experiments) in breeding birds have yielded conflicting results. Some studies report no effect of food supplementation on parental provisioning, offspring growth or offspring survival, some report positive effects, and others report negative effects (reviewed in [2,3,6–9]).

In species that provide parental care to dependent young, parents must balance the allocation of time and energy in the care provided to their current offspring against time and energy invested in self-care and future reproduction [10,11]. Optimal allocation is mediated by the relative costs and benefits of a given level of investment [11,12]. Parents can increase the growth and survival of their current offspring by investing more in parental care behaviours, such as provisioning [13–19]. However, parents that invest more heavily in current offspring may reduce their capacity to invest in future offspring, because increased investment in current offspring comes at the cost of self-care [20,21]. Thus, when additional food is available (either naturally or via food supplementation), parents may not necessarily increase the rate at which they provide food to their current offspring, but may instead favour allocation towards self-care, which may account for some of the conflicting results previously reported (reviewed in [2,3,6–9]).

Specifically, we suggest that the effect of food supplementation on parental investment in the current brood may fit into one of three (non-exclusive) models that differ in how parents alter investment in current versus future reproduction with increasing food availability. We term these ‘additive’, ‘substitution’ or ‘insurance’ models. First, food-supplemented parents may take advantage of increased food availability to increase the level of care provided to young [2,3], which we call the ‘additive model’. Under this scenario, food supplementation of provisioning adults means that even if parents invest the same effort (time and/or energy) in searching for food, parents will be able to provision young at a higher rate due to a higher encounter rate with suitable prey under supplementation. This may be particularly true in species where parents cache food items (e.g. [3]), because supplemental food can be cached nearby, eliminating the need to search for prey, at least until the cache is depleted. This could allow for higher prey delivery rates without any negative effect on parental condition and/or future reproduction. If parents adopt an additive strategy when provided with supplemental food, this should result in an increase in provisioning rate, nestling body masses and survival of offspring at supplemented nests relative to controls (table 1).

**Table 1.** Summary of the key predictions of each proposed model of provisioning: additive, substitution and insurance. All predictions refer to supplemented nests relative to control nests. Although we did not have data available to test predictions related to parental survival and future reproduction, we have included them here for completeness (text marked in italics). Predictions that were supported by our analyses are in bold font.

metric	additive	substitution	insurance
nestling body mass	**increase**	no change	**increase in mean, decrease in variance**
nestling survival	**increase**	no change	**increase in mean**
provisioning rate	increase	**no change**	increase in mean, **decrease in variance**
*parental survival*	*no change*	*increase*	*no change*
*future reproduction*	*no change*	*increase*	*no change*

Alternatively, food-supplemented parents may take advantage of food supplementation to increase investment in self-care, which we term the ‘substitution model’ (see also [2]). The substitution model predicts that food supplementation enables breeding adults to maintain the same level of care to their young (e.g. rate of energy delivery) with less effort (e.g. less time spent searching for food), allowing them to reallocate time and/or energy towards self-maintenance. Thus, under the substitution model, we would predict comparable provisioning rates, nestling body masses and survival at supplemented nests relative to controls (table 1). The increased investment in self-care also means that parents that adopt a substitution model when they are food supplemented would be predicted to experience increased survival and future reproductive success (table 1).

Finally, food supplementation may provide a buffer against variables that lead to yearly variation in reproductive success, such as years with low food availability and/or higher energetic costs, such that food supplementation allows breeding adults to mitigate the negative effects of such events on their offspring [22]. We term this the ‘insurance model’. Under this model, the effects of supplementation should be stronger when background food availability is low compared with when it is high (e.g. [2,3,22]). Provisioning rate may be either unchanged or increase if parents adopt the insurance model; if background prey availability is high, provisioning rates may be unaffected by food supplementation, while low background prey availability may result in increased mean provisioning rates for supplemented parents, relative to control parents. When looking across breeding attempts that include years with both high and low background prey availability, this would mean that supplemented nests should experience higher average provisioning rates, and lower variance in provisioning rates compared with control (non-supplemented) nests (table 1). Previous work in the black sparrowhawk (*Accipiter melaoleucus*) has shown that for a given provisioning rate, greater consistency in prey deliveries (i.e. lower variance) leads to improved survival of offspring [23]. Since the regular supply of supplemental food should provide a buffer against stochastic variability in background levels of food availability facilitating greater consistency in prey delivery, under the insurance model, food-supplemented parents should have lower inter-annual variation in offspring production and be able to produce offspring with a higher body mass when background food availability is low compared with parents at non-supplemented nests. Finally, under the insurance model, because parents can successfully fledge young under a wider range of environmental conditions, we would expect to observe an overall increase in nestling survival probability.

In the present study, we conducted a food supplementation experiment with Arctic-breeding peregrine falcons (*Falco peregrinus tundrius*) across five successive breeding seasons (2013–2017) to evaluate support for each of the three models described above. Specifically, we aimed to evaluate how parental food supplementation influences (i) mean and variance in provisioning behaviour and (ii) mean and variance in measures of reproductive success (nestling survival and nestling body mass) to test the predictions laid out in table 1. Although the three models also generate predictions about parental survival and future reproduction (table 1, italicized rows), we did not have a sufficient fraction of our study population marked to allow us to track individuals across years (see §2). Therefore, our analyses were restricted to the effects of food supplementation on current reproduction. Understanding the potential effects of food supplementation on parental investment will provide valuable insights into the trade-offs that provisioning peregrines face and the mechanisms they use to maximize their reproductive success.

## 2. Methods

### 2.1. Field methods

#### 2.1.1. Study site and population

This study was conducted in Rankin Inlet, Nunavut (62°812′ S, −92°094′ E), an area comprising large tundra stretches and rocky outcrops on which peregrine falcons nest. Peregrines included in this study nested across coastal, island and mainland sites. A total of 127 nests were monitored across the 5-year study period (range 18–25 nests per year). However, sample sizes included in analyses varied for different response variables. An overview of sample sizes is provided in table 2, and electronic supplementary material, table S1 provides detailed justification for the removal of some nests from specific analyses. Although we did not collect data on natural prey availability as part of our study, previous work in the same population has shown that there is a large inter-annual variation in peregrine prey densities [24,25]. We, therefore, assume that the 5-year study period included here was sufficiently long to capture both years of high and low relative prey abundance.

**Table 2.** Overview of sample sizes as a function of treatment (C = control or S = supplemented) for each trait and each year, as well as overall sample sizes for the 5-year study. All sample sizes refer to the number of nests, except for sample sizes provided in parentheses, which refer to the number of nestlings.

	parental care						reproductive success					
	**total nests studied**			**provisioning data**			**nestling body mass** **nests (nestlings**)			**nestling survival** **nests (chicks**)		
**year**	**C**	**S**	**total**	**C**	**S**	**total**	**C**	**S**	**total**	**C**	**S**	**total**
2013	17	8	25	11	1	12	17 (33)	8 (24)	25 (57)	17 (55)	8 (28)	25 (83)
2014	13	10	23	11	8	19	13 (21)	10 (25)	23 (46)	13 (43)	10 (36)	23 (79)
2015	11	10	21	11	10	21	11 (16)	10 (28)	21 (44)	11 (35)	10 (30)	21 (65)
2016	17	12	29	16	12	28	15 (22)	12 (30)	27 (52)	15 (51)	12 (38)	27 (89)
2017	17	12	29	17	12	29	17 (25)	12 (25)	29 (50)	17 (46)	12 (43)	29 (89)
overall	75	52	127	66	43	109	73 (117)	52 (132)	125 (249)	73 (230)	52 (175)	125 (405)

Egg laying took place between early and late June each year, with females each laying between two and four eggs [26]. Eggs are incubated primarily by females for 33.5 days on average [27,28] resulting in (asynchronous) hatching of between one and four nestlings in July [26]. In this study, the average hatch date was 13 July (range: 5−26 July). Peregrines are a caching species [29], meaning that they do not need to use supplemental food immediately, but may cache it for later use if and when required. Peregrines are generalist predators with a broad prey range including mammals, songbirds, shorebirds and waterfowl. Our study population in particular has a high contribution of mammalian prey in the diet relative to other populations of peregrines, with mammalian prey comprising up to one-third of the total diet [30,31].

#### 2.1.2. Supplementation experiment

Within our study area, several nest sites are accessible only by helicopter, or have very limited accessibility depending on the weather. As these sites could not be visited regularly, they were excluded from the supplementation experiments (see electronic supplementary material, table S2). Of the nests that were accessible and therefore could be visited regularly, they were alternately assigned to supplemented or control treatments as the nests hatched. Treatments were assigned using a blocked spatial design such that there were 1–2 control nests in close proximity to each supplemented nest. Each year, 8–12 nests were food supplemented, which resulted in a total of 52 nests that were food supplemented (see table 2 for sample sizes per treatment group and year) and 75 nests that were control nests. Both supplemented and control nests were visited at the same frequency; every 5 days, weather permitting, to control for rates of human disturbance at nests between supplemented and control nests.

Supplemented broods received commercially produced common quail (*Coturnix coturnix*) except in 2014 when there were four instances (out of 55 supplementations in that study year) where supplemented broods received commercially produced rock pigeons (*Columba livia*) due to a lack of availability of quail. In these cases, pigeon was substituted 1 : 1 for quail according to the required supplementation treatment for the nests (see electronic supplementary material, table S3). Supplemental food was stored frozen and thawed to outside temperature prior to nest delivery. Supplemental quails or pigeons were provided whole (feathered) and placed within 1 m of the scrape. Quails were selected as supplemental food based both on their commercial availability and because they have a similar nutritional profile to a number of other small birds and mammals [32], suggesting they are probably of similar nutritional quality to naturally available prey in the study area.

Supplementation occurred between the nestling age of 5 to 25 days. We waited until nestlings were 5 days of age to begin supplementation to avoid disturbance immediately after hatch, and we stopped supplementation at age 25 days to avoid inducing early fledging. Furthermore, nestling body mass begins to asymptote around day 25, suggesting that supplementation beyond that point might have limited effect [33]. We also avoided visiting nests during inclement weather conditions such as during heavy rain, again to reduce unnecessary disturbance. When visits were missed, we compensated by providing supplemented nests with the missed quantity of quail in addition to the scheduled amount during the subsequently planned nest visit, or, where possible, by delivering the intended quantity of quail on the following day. The quantity of quail provided corresponded to approximately 50% of the brood’s age-specific energetic demand. Such demand was derived at the individual level from the observed amount of food necessary to suppress begging among captive bred falcon nestlings (L Oliphant 14 May 2013, personal communication). This resulted in increasing quantities of quail being provided to larger broods and as nestlings grew older (see electronic supplementary material, table S3 for a detailed record of food supplementation quantities as a function of brood size and age). Although each visit resulted in deposits of large amounts of food, we expected that peregrines would exhibit normal caching behaviour [34], and that supplemented quail could therefore be rationed over the following days. During our initial visits, we observed adults from a blind to confirm that they cached the quail in nearby locations and that supplemental food was being utilized only by adults at the supplemented nests. Although we were not able to subsequently monitor the caches to confirm that they were not used by other, non-target, pairs of peregrines, this is unlikely given that peregrines are highly territorial and aggressively defend their territory against intruders [35]. Across the study duration, there were only two instances where untouched quail remained at active nest scrapes upon our return 5 days later, once in 2014 and once in 2016.

#### 2.1.3. Data collection and processing

We began visiting historical nest sites in May of each study year, during the arrival of peregrines at the study site. We placed motion-sensitive cameras within 1 m of all active nests that collected images of all nest visits made by adults, including during periods of low light. Active nests were visited every 5 days, weather permitting (as previously discussed). The hatch date was determined from nest camera images. Hatched nestlings were marked on the right thigh with non-toxic markers as red, green or blue, or left unmarked, to allow individuals to be monitored for the duration of the breeding season. We weighed nestlings at each visit to the nearest gram. We used the last body mass measurement taken in our analyses of nestling body mass at assumed fledging. For more details on field protocols, see McKinnon *et al*. [36].

Nestlings that were still alive on the final nest visit were presumed to have survived to fledging. Final nest visits occurred between nestling ages 21 and 35 days. We did not visit nests after day 35 to avoid inducing early fledging, however, not all nests were visited at nestling age 35 days due to logistical constraints such as inclement weather limiting access to some nest sites. Nestlings that were alive on the final nest visit were leg banded for identification purposes. Avian leg gauges from Avinet Research Supplies were used to measure the nestlings’ leg diameter, and appropriately sized leg bands were subsequently applied. Sex determination of nestlings followed the guidelines outlined in the banding guide provided by the United States Geological Survey (USGS) (available at https://www.pwrc.usgs.gov/BBL/Bander_Portal/login/speclist.php). Nestlings fitted with a band size of 6 (or smaller) were designated as male, while those with a band size of 7A (or larger) were identified as female. A total of 243 nestlings were sexed via this method: 114 females and 129 males. An additional six nestlings were not banded or sexed because it was deemed that banding these nestlings might induce early fledging based on the size and behaviour of the nestlings during the banding visit. These six nestlings were removed from the analysis of body mass but were retained for all other analyses where sex was not included in the models (see §2.2., Statistical methods).

Provisioning data were extracted from time-stamped nest camera images and were analysed at the level of the pair, because peregrines exhibit a division of labour such that most hunting is done by the male, but most provisioning to young is done by the female [37]. Further details of extraction and processing of provisioning data can be found elsewhere [36]. We calculated inter-visit intervals (IVIs) as the time (in min) between the beginning of successive provisioning visits. Between late July and mid-August (the period in which we collected provisioning data), our study area did not experience full darkness. As such, provisioning visits could (and did) happen at any hour within the 24 h day, and therefore we did not treat successive provisioning visits any differently if they occurred within the same day or across days. Hatch day (i.e. nestling age 0) was excluded from analysis, given the possibility for great variation in opportunity for feeding generated by the time of hatching in the day (for example, the feeding opportunities of a nestling born in the morning versus late evening). Provisioning behaviour was only analysed between nestling day 5 (i.e. the start of nest supplementation experiments) up to a nestling age of 12 days. We did not include camera trap data collected after the nestling age of 12 days because we observed the first instance of a nestling moving out of frame of the nest camera at age 13 days, meaning beyond this age we could not be confident camera traps were capturing all parental provisioning visits. There were few nests at which cameras were placed but extraction of provisioning data was not possible due, for example, to camera and memory card problems; see electronic supplementary material, table S1 for full details of which nests were excluded and why.

Although the three models laid out in table 1 include predictions about parental survival and future reproduction, we were unable to obtain the data required to test these predictions. This is because logistical constraints and variation in catching success meant that only approximately 50% of adults in the population in any given year are banded. Thus, we were unable to track individuals across years to evaluate the effect of supplementation in one year on survival and/or reproduction in the next.

### 2.2. Statistical methods

We first evaluated that the pseudo-randomized assignment of supplemented and control nests did not result in any significant differences in hatch date, clutch size or number hatched across treatments. To do this, we constructed three Bayesian regression models (i.e. one each for hatch date, clutch size and number hatched) using Hamiltonian Monte Carlo (HMC) sampling conducted using the ‘brm’ function from the ‘brms’ package [38] in R (v. 4.2.0). All three models included treatment as a fixed effect, with year and nest site ID included as random effects to account for inter-annual variation in breeding parameters, and non-independence of nests at the same sites across multiple years. The hatch date was modelled based on a Gaussian error distribution, while the number hatched and clutch size were count data, and so modelled with a Poisson error distribution.

Next, we developed three models, again using the ‘brms’ package for Bayesian regression modelling, to test the predictions laid out in table 1. All models included fixed and random effects. First, we modelled (log-transformed) IVI with a Gaussian error distribution and Markov chain Monte Carlo (MCMC) sampling using two different model structures. Both models included fixed effects for treatment (‘food supplemented’ or ‘control’), hatch date (centred and scaled at the level of the full dataset) and nestling age (centred and scaled at the level of the full dataset), and random effects for site, pair and year. We also modelled heterogeneous residual variance (sigma) as a function of treatment within this model. However, we ran a second version of this model keeping all variables the same except including brood size on the day of observation (centred and scaled at the level of the full dataset) as an additional fixed effect. By including models both without and with brood size as a covariate, we were able to look at total parental effort as a function of treatment (regardless of any treatment-related differences in brood size), as well as treatment-related differences in parental effort experienced per nestling, respectively.

We modelled nestling survival (i.e. survival to the final nest check), a measure of reproductive success, using logistic regression with a Bernoulli distribution and MCMC sampling. The Bernoulli distribution models the probability of success (survival) as a function of the predictors. For this model, all individuals that hatched were included and coded as either 1 (survived to the final nest visit) or 0 (did not survive to the final nest visit). This model included fixed effects for treatment, number hatched (centred and scaled at the level of the full dataset), and hatch date (centred and scaled at the level of the full dataset) and random effects for site, pair and year.

Finally, we modelled the nestling final observed body mass (grams) with a Gaussian error distribution and MCMC sampling. This model included fixed effects for treatment, number of nestlings that survived to final nest check (see above) (centred and scaled at the level of the full dataset), hatch date (centred and scaled at the level of the full dataset), nestling age (centred and scaled at the level of the full dataset) and nestling sex (male or female), and random effects for site, pair and year. Nestling age was included because the final nestling body masses obtained range from day 21 post-hatch to day 35 post-hatch. During this period, growth is approximately linear until nestlings reach their asymptotic mass [33]. Thus, we included nestling age as a covariate to account for the age gradient in final mass measurements. We included nestling sex as peregrines are sexually size dimorphic, with females being both larger and heavier than males [39]. Sex was coded as −1 or +1 such that the model intercept was estimated for the average nestling, irrespective of sex, as our predictions did not necessitate an evaluation of the interaction between treatment and offspring sex.

For each model, the mode of estimated effects (β, σ or ρ) and 95% credible intervals (CrI) are presented and used to evaluate support for each effect. Estimates with CrIs that do not overlap zero are interpreted as providing strong support. Estimates with CrIs centred around zero are interpreted as providing no support for the effect. For estimates that were biased away from zero, but whose 95% CrI overlapped zero, we calculated the proportion of the posterior distribution that was in the opposite direction of the mean estimate value calculated from all model iterations. We present these, where appropriate, for the fixed effects in our models, within the results statement, as ‘pr’. When CrIs overlapped zero by less than 15%, we interpret this as providing moderate support for an effect in the estimated direction because this equates to over five times greater support for the interpretation of an effect in the estimated direction relative to an effect in the opposite direction [40].

## 3. Results

We first confirmed that the assignment of supplemental and control nests was random with respect to hatch date, clutch size or number hatched. Our analysis revealed no support for a difference between supplemented and control nests in hatch date (−0.42, 95% CrIs = −1.28, 0.45; pr = 0.168), clutch size (0.00, 95% CrIs = −0.15, 0.14) or number hatched (0.05, 95% CrIs = −0.10, 0.21; pr = 0.237).

### 3.1. Provisioning effort (inter-visit intervals)

**Figure 1.**
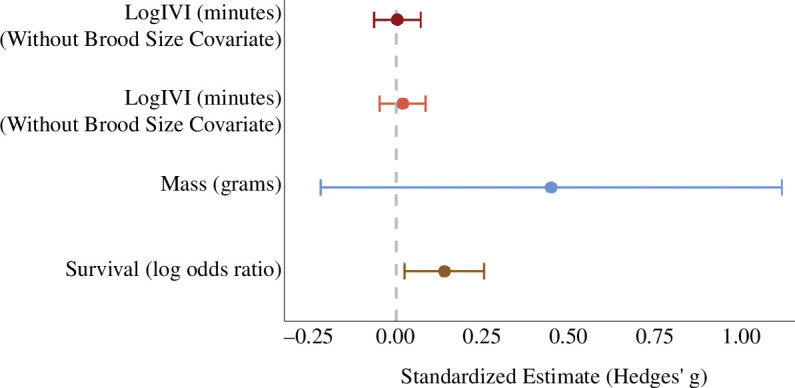
Estimated effect of food supplementation on parental provisioning rate (log IVI (min)), nestling survival (log odds ratio) and nestling body mass at the final nest check (grams). Estimates and 95% CIs have been standardized (reported as Hedges’ g) to facilitate comparison across response variables with different measurement units. Estimates are calculated from model outputs presented in table 3. Positive estimates indicate that providing supplemental food resulted in an increase in the response variable.

We observed 5423 provisioning visits at 109 nests across our 5-year study period, from nestling ages 5 to 12 days, reflecting the period of supplementation. The average number of daily provisioning visits per nest was 6.81 (range 1 to 17). We did not observe a significant effect of food supplementation on mean IVI in either the IVI model excluding brood size (β = 0.01, 95% CrIs = −0.12, 0.14; pr = 0.41, table 3, figure 1) or the IVI model including brood size (β = 0.05, 95% CrIs = −0.08, 0.17; pr = 0.24; table 3, figure 1). However, supplemental feeding was associated with reduced variance in provisioning IVI both for the model without brood size covariate (σ = −0.04, 95% CrIs = −0.08, 0.00) and the model with brood size covariate (σ = −0.04, 95% CrI = −0.08, 0.00). Additionally, the log IVI of provisioning decreased with increasing brood size (β = −0.30, 95% CrIs = −0.44,−0.16) and with increasing nestling age (without brood size covariate: β = −0.11, 95% CrIs = −0.13,−0.09; with brood size covariate: β = −0.12, 95% CrIs = −0.14,−0.09). We also found strong support that log IVI varied across breeding pairs, territories and years (see table 3). However, we found no support for an effect of hatch date on IVI (see table 3).

**Table 3.** Model results for provisioning behaviour (log IVI (min)), nestling survival (survived = 1, died = 0) and nestling body mass (g) at the final nest visit. Two models exploring sources of variation in provisioning behaviour are presented, one without brood size as a covariate and one with brood size as a covariate to differentiate between total provisioning effort independent of treatment-related differences in brood size and provisioning effort per nestling, respectively. Provisioning behaviour and body mass were modelled with Gaussian error distributions, and as such, we were able to model treatment-related differences in residual variance. Survival probability was modelled with a Bernoulli error family, which assumes a residual variance of 1, and therefore, does not allow for modelling treatment-related differences in residual variance. Significant fixed effects and heterogeneous residual variances are indicated in bold.

	log IVI (min) (not controlling for brood size)	log IVI (min) (controlling for brood size)	nestling survival	nestling body mass (g)
fixed effects	**β (95% CI**)	**β (95% CI**)	**β (95% CI**)	**β (95% CI**)
intercept	**4.94 (4.66, 5.23**)	**4.91 (4.64, 5.19**)	**−0.06 (−1.31, 1.11**)	**636.43 (554.97, 713.31**)
hatch date	−0.01 (−0.05, 0.03)	−0.02 (−0.06, 0.02)	**−0.57(−1.03,−0.22**)	−3.53 (−11.22, 4.18)
number hatched	NA	NA	−1.44 (−3.23, 0.17)	NA
brood size	NA	**−0.30(−0.44,−0.16**)	NA	−14.09 (−44.22, 15.88)
nestling age	**−0.11(−0.13,−0.09**)	**−0.12(−0.14,−0.09**)	NA	**16.09 (−5.83, 26.17**)
treatment	0.01 (−0.12, 0.14)	0.05 (−0.08, 0.17)	**2.39 (1.11, 3.93**)	24.68 (−3.53, 53.22)
random effects	**σ (95% CI**)	**σ (95% CI**)	**σ (95% CI**)	**σ (95% CI**)
site ID (i.e. territory)	0.09 (0.00, 0.20)	0.07 (0.00, 0.17)	0.71 (0.04, 1.75)	22.36 (1.92, 43.52)
pair ID	0.28 (0.22, 0.35)	0.27 (0.21, 0.33)	2.59 (1.70, 3.79)	33.45 (12.16, 52.27)
year	0.25 (0.07, 0.71)	0.26 (0.07, 0.70)	0.90 (0.05, 2.63)	36.32 (9.23, 93.56)
residual variance intercept	0.05 (0.02, 0.08)	0.05 (0.02, 0.08)	1 (1,1)^a^	4.84 (4.67, 5.01)
residual variance (treatment)	**−0.04 (−0.08, 0.00**)	−**0.04 (−0.08, 0.00**)	NA	**−0.76(−0.98,−0.54**)

^a^ Residual variance fixed to 1 for binomial error distribution.

### 3.2. Offspring survival probability

Our analysis was based on the survival outcomes of 405 nestlings at 125 nests across the 5-year study duration. Overall, 156 nestlings died before the final nest check, and 249 survived. We found strong support for a negative association between nestling survival probability and hatch date (β = −0.57, 95% CrIs = −1.03,−0.22) and strong support that supplemental feeding improved survival probability (β = 2.39, 95% CrIs = 1.11, 3.93; figure 1). We found moderate support that survival probability decreased with the increasing number of young hatched in a given nest (β = −1.44, 95% CrIs = −3.23, 0.17; pr = 0.036). Additionally, we found strong support for variability in survival probability across breeding pairs (σ = 2.59, 95% CrIs = 1.70, 3.79), territories (σ = 0.71, 95% CrIs = 0.04, 1.75) and years (σ = 0.90, 95% CrIs = 0.05, 2.63) (see also table 3).

### 3.3. Nestling body mass

We analysed nestling body mass for the 249 nestlings from 102 nests that survived to the final nest check. The average nestling mass, irrespective of sex, was 618.96 g (95% CrIs = 558.54, 676.41). Nestling body mass was significantly higher for female nestlings compared with males (β = 104.40, 95% CrIs = 95.23, 113.33) irrespective of treatment group, as expected given that the body size was used as a criterion to assign nestling sex. We found moderate support that supplemental feeding increased mean nestling body mass (β = 24.68, 95% CrIs = −3.53, 53.22; pr = 0.035; figure 1) and strong support that supplementation reduced variability in nestling body mass (σ = −0.76, 95% CrIs = −0.98, −0.54) compared with controls. Additionally, we found that age at final body mass measurement significantly impacted body mass, with older nestlings having a higher mass on average (β = 16.09, 95% CrIs = 5.83, 26.17; pr = 0.001). However, there was no support that later hatch dates were associated with lower nestling body masses (β = −3.53, 95% CrIs = −11.22, 4.18; pr = 0.224). Similarly, there was no support that larger brood sizes (at the final nest visit) resulted in lower nestling body mass (β = −14.09, 95% CrIs = −44.22, 15.88; pr = 0.177). Finally, we found strong support for differences in nestling body mass across pairs (σ = 33.45, 95% CrIs = 12.16, 52.27), territories (σ = 22.36, 95% CrIs = 1.92, 43.52) and years (σ = 36.32, 95% CrIs = 9.23, 93.56).

## 4. Discussion

We evaluated the effect of parental food supplementation on provisioning behaviour and reproductive success in Arctic-breeding peregrine falcons. We assessed results in view of three potential non-exclusive strategies parents may adopt when provided with supplemental food: additive, substitution or insurance (table 1). We found partial support for all three mechanisms. Consistent with both the additive and insurance models, we found strong support that supplementation increased the probability of nestling survival and moderate support for an increase in nestling body mass. We also observed decreased variance in nestling body mass and decreased variance in provisioning IVIs under food supplementation, consistent with the insurance model. However, we found no support for a change in the mean provisioning rate (provisioning IVIs). This is consistent with the substitution model, but inconsistent with either additive or insurance models. This study contributes to existing knowledge of the effects of food supplementation on parental investment. Importantly, it also demonstrates that none of the models as laid out in table 1, on their own, can fully account for the responses to food supplementation observed in the present study. Below, we discuss how our results might be explained by a combination of additive, substitution and insurance models, and how future studies could test our suggested hybrid model.

If peregrines use food provided during supplementation experiments either in addition to their baseline parental investment (‘additive model’; [2,3]) or specifically to meet offspring demand under more challenging conditions (‘insurance model’; [22]), we predicted an increase in mean provisioning rates (i.e. decrease in provisioning IVIs) for peregrines that received the supplementation treatment. We found no support for this prediction. Mean IVIs did not differ as a function of treatment; however, we did find support for reduced variance in IVIs for food-supplemented nests. There are several potential explanations for this finding: (i) peregrines exhibit changes in prey selectivity in response to food supplementation, (ii) lack of effect is due to incomplete provisioning data, and/or (iii) lack of statistical power. We discuss each of these in turn, below.

First, our findings may indicate that rather than reducing IVI, supplementation allowed parents to change prey selectivity to favour more nutritionally or energetically beneficial prey types and/or select for larger prey items. Shifts in prey type could explain the increase in nestling body mass and survival in the absence of an increase in provisioning rate (e.g. [41]), and could also be associated with reduced variance if prey types differ in the variance in encounter rate [42]. Initially, we aimed to collect prey type and biomass information from our camera trap images. However, around 30% of provisioning visits lacked accurate data due to issues such as poor image quality and partially obstructed views of prey. This is not only a large fraction of missing data, but missing data are likely to be non-random. For example, small prey items are likely to be more easily missed in camera trap images compared with larger prey. Moreover, our nest cameras only captured images of the scrape, missing details about surrounding areas where prey might have been processed. Consequently, incomplete and potentially biased prey type and size data were excluded from further analysis. However, we acknowledge that shifts in prey selectivity may have occurred and could account both for the higher survival and increased nestling body mass observed at supplemented nests in the absence of changes in provisioning rates. Accurate measures of prey type and quality provisioned to young would be required to test this directly.

Second, our analysis of provisioning data was restricted to between nestling days 5 and 12, which may have failed to capture the relevant period in which shifts in parental provisioning in response to food supplementation could be observed. As nestlings age, their demand for food increases [36]. We found this to be true even within the 12-day period, thus the increase in demand and the resultant increase in sibling competition later in the breeding season may result in more pronounced differences in mean IVIs across supplemented in control nests. However, previous research indicates that peregrine growth (and thus potential increases in demand) plateaus at around day 25 post-hatch [33], indicating we captured a large fraction of the period of increasing demand (7 days out of 25 days, or greater than 25%). This, coupled with our finding of an effect size of almost 0 between days 5 and 12 (β = 0.01, 95% CIs = −0.05, 0.03), suggests that it is unlikely that significant differences in provisioning rate at supplemented and control nests would emerge only in the latter half of the breeding season.

Third, the lack of effect of food supplementation treatment on mean IVIs may be because we lacked statistical power to detect effects. This is unlikely given that our estimates of provisioning effort were based on 5423 observations at 109 nests over 5 years, suggesting that our dataset should have had sufficient power to detect an effect of supplementation treatment on provisioning IVI had it been there. Indeed, we were able to detect significant effects of nestling age and brood size, two proxies for brood demand, on provisioning IVI. Consistent with a large body of earlier empirical work in both peregrines (e.g. [36,43–45]) and birds in general (e.g. [19,46–48]), peregrines decreased provisioning IVIs with increasing brood demand. Furthermore, we observed a significant decrease in the variance in IVI in supplemented nests (table 3). Detecting heterogeneous residual variances is notoriously data hungry [49], thus, the fact that we had the power to detect this effect suggests that the lack of observed effect of treatment on mean IVIs reported here is likely to be biologically real and not due to lack of statistical power.

We suggest that the lack of observed effect of food supplementation treatment on mean IVI is biologically real and not due to the lack of complete provisioning data (limited to days 5–12) or low statistical power. While this observation is consistent with the substitution model, our findings that food supplementation resulted in increased nestling survival and increased nestling body mass suggest that the substitution model, as we originally proposed it, cannot fully account for our results (see table 1). Overall, our data are consistent with food-supplemented peregrines adopting a combination of the three models. The decreased variance in IVI observed in food-supplemented nests is at least partially consistent with the insurance model, and decreased variance may account for the increased survival and the increased body mass of the young of food-supplemented nests. For example, previous work on another raptor, the black sparrowhawk, found that even when mean provisioning rates were identical, lower variance in provisioning was associated with increased survival [23].

Another possibility is that our view of expected changes under the substitution and additive models may have been unduly limited. In the substitution model, we initially only considered substitution within the same form of parental care (i.e. provisioning behaviour); however, substitution may be occurring across different forms of parental care, specifically from provisioning to brooding and/or nest defence. In this case, parents could facilitate an increase in offspring survival without a change in provisioning effort, which is consistent with our findings. For example, since peregrines are a caching species [29,50], the provision of supplemental food allows them to increase their reserve food supply in caches located near the nest scrape, meaning parents can more actively shelter and protect their offspring from both inclement weather and predation, both of which could have contributed to the increased survival likelihood at food-supplemented nests. Increased nest attendance would be expected to be particularly important in our study population, as previous work has shown that inclement weather is a major contributor to nestling mortality, and furthermore, that sheltering young from rainfall leads to a significant increase in survival [28]. We suggest that food-supplemented broods may benefit from higher nest attendance by parents, which is a readily testable prediction in a variety of systems but would require data on non-provisioning nest visits in addition to provisioning nest visits. Thus, we suggest that our findings of increased nestling survival and nestling body mass in supplemented nests, despite constant IVI, may indicate that parents are using food supplementation to add to their total level of care provided to offspring by substituting effort that would have otherwise been spent on searching for prey, towards effort spent on nest defence and protection of offspring. This equates to a hybrid of our originally proposed models. Furthermore, it is worth noting that we were unable to test predictions for any of the three models related to parental survival and/or future reproduction. Thus, it is also possible that parents adopted a combination of insurance, increased allocation to other forms of parental care (e.g. brooding, a modified version of the additive model) as well as increased investment in self-care (substitution model) when provided with supplemental food.

Our study provides important insights into the response of provisioning peregrine falcons to increases in food availability and the resultant impact on offspring both in terms of nestling body mass and nestling survival. We found strong support for increased survival and moderate support for increased body mass of nestlings at food-supplemented nests, with no evidence of changes in mean parental provisioning rates. Given the non-mutually exclusive nature of the three models we proposed, future studies would allow for stronger inference if they tested the full suite of predictions derived from these models (table 1), including parental survival and future reproduction. For example, if well-powered studies fail to detect any effect of food supplementation of parental survival and future reproduction, and also observe increased nest attendance, that would provide strong support for the proposed model whereby supplemented parents increase investment in other (non-provisioning) forms of parental care. Detailed observation of the type and size of prey, as well as provisioning decisions (e.g. evenness of provisioning among young) would also provide important insights into the mechanisms underlying the observed changes in nestling body mass and survival. Understanding the potential impacts of food supplementation on parental investment is crucial for gaining insights into the trade-offs and mechanisms employed by provisioning peregrine falcons (and other species) to maximize reproductive success.

## Data Availability

All data and code required to reproduce the analyses and figures presented in the manuscript are archived on Dryad [51]. Supplementary material is available online [52].
